# Book Reviews

**Published:** 1799-08

**Authors:** 


### CRITICAL RETROSPECT OF MEDICAL AND PHYSICAL LITERATURE.

### [FOREIGN AND DOMESTIC.]

*Remarks on ſome of the Opinions of the late Mr.* JOHN HUNTER, *reſpecting the Venereal Diſeaſe; in a Letter to* JOSEPH ADAMS, *M. D. By* HENRY CLUTTERBUCK, *Surgeon.* 8vo. pp. 72. 1s. 6d. *London.* Booſey.

THE author ſtates the opinions of Mr. Hunter, which he propoſes to examine in this manner, viz.

"The following, I believe, are the chief points of Dr. Hunter's doctrine of the venereal diſeaſe.

"1. That the venereal poiſon, being taken into the ſyſtem, becomes univerſally diffuſed, and contaminates at once, all the parts which are ſuſceptible of the venereal action; and that it is ſoon afterwards expelled the ſyſtem, along with ſome or other of the excretions.

"2. That the parts contaminated do not immediately go into venereal action; but that they acquire a new ſtate or condition, and which is termed by him, a *diſpoſition to take on the venereal action.*

"3. That *diſpoſition* once formed in a part, neceſſarily goes on at ſome future period, to *action.*

"4. That mercury cures *venereal action*, but does not remove the *diſpoſition* previouſly formed, and which is not yet come into action.

"5. That although mercury does not deſtroy the diſpoſition already formed, yet that it prevents it from forming.

"6. That although the diſpoſition continues, it does not go into action during the uſe of mercury.

"7. That the action having once taken place, goes on increaſing without wearing itſelf out.

"8. That parts once cured never become again contaminated from the ſame ſtock of infection.

"9. That the matter of the ſecondary ulcers is not infectious.

"10. That the venereal action is as ſoon deſtroyed in a large chancre as in a ſmall one, the mercury acting equally in all its parts.

"Theſe are the outlines of a doctrine, which, by its ingenuity and originality, has deſervedly attracted a very conſiderable ſhare of the public attention, and laid the foundation of a practice, in many reſpects different from that which had before generally prevailed.

The firſt opinion which our author examines is, the diſtinction between *diſpoſition* to venereal action, and the *action itſelf.* If this opinion had been merely connected with the ſpeculative pathology of ſyphilis, it would probably have paſſed unnoticed; but when the very important practical inference is drawn from it, by ſo high an authority as that of Mr. Hunter, viz. *that it is the actual diſeaſe alone which is curable, and not the diſpoſition*, it becomes of importance to inquire into its truth.

Mr. Clutterbuck combats this opinion, on the ground that the contamination produced by the poiſon, called *diſpoſition*, is actual diſeaſe, though not obviouſly ſyphilis: and ſince diſeaſes are more eaſily cured in their commencement, or during their formation, than when formed, he would conclude, *a priori*,

*priori*, that the *dispofition* is at leaft as curable as the difeafe. This, however, is only oppofing opinion to opinion. Mr. Clutterbuck next proceeds to ftate the arguments and experiments which fupport his fide of the queftion. Our limits do not permit us to give long extracts, but the following argument appears to us to carry much weight with it.

The parts of the body fucceffively attacked are commonly in this order; viz. the throat, the fkin, the bones. Now as all thefe are allowed by Mr. Hunter to be contaminated at the fame time when the virus is abforbed, it ought to follow that each of thefe parts will in every inftance become affected, which is contrary to experience: for, according to Mr. Hunter's doctrine, if the patient applies mercury on the appearance of a venereal fore throat, while there are no other fymptoms, he can only expect to cure the throat, but the blotches and nodes being only fufpended for a time, will certainly appear afterwards, when the mercurial action ceafes. As this, however, is far from being always the cafe, we muft infer that mercury cures the *difpofition* to blotches and nodes, as well as the ulcers of the throat.

In a fubfequent part of the pamphlet, Mr. Clutterbuck adduces feveral facts in oppofition to Mr. Hunter, refpecting the innocence of the matter of fecondary ulcers; the difeafe wearing itfelf out, &c.

The work contains a number of inftructive cafes, and many practical obfervations on the treatment of lues, well worthy the attention of practitioners.

---

*A Cafe of Diabetes, with an hiftorical fketch of that difeafe: By* THOMAS GIRDELSTONE, *M. D.* 8vo. 112. pp. (Price three fhillings) 1799. *London*, Robinfons.

In the preface to this work, the author acknowledges the obligations he owes to Dr. Rollo, and Mr. Cruikfhanks, for the annexed cafe of Diabetes.

"But" fays he," amidft the multiplicity of the correfpondents of Dr. Rollo, he feems, by his publication on diabetes, to have overlooked fome parts of my letters to him, concerning my former patient Capt. M., and certainly did not rightly underftand in what manner Capt. M. had been for three months under the care of a furgeon and phyfician, *without the circumftance of increafed urine being known* to them; it is but juftice to Mr. Penrice, the furgeon, and myfelf, that that part of the retrofpect of Capt. M's cafe, which has been omitted by Dr. Rollo, fhould precede the detail of my prefent diabetic cafe." p. ii.

Subfequent to this intimation Dr. Girdelftone gives a concife ftatement of the treatment adopted by Mr. Penrice, as well as himfelf, together with the preceding fymptoms, and the gradual progrefs of the difeafe, in the cafe of Capt. M. Our limits do not admit of inferting the interefting particulars ftated in the preface relative to the afore-mentioned cafe, as well as that of another patient, who was likewife cured by a ftrict adherence to animal diet.

The author has given an hiftorical fketch of diabetes, together with an account of the various remedies prefcribed by phyfians, from Aretæus, Galen, and Avicenna, down to the prefent day. He has interfperfed this fketch with a variety of claffical and appofite quotations. After having reviewed the theories of this difeafe, maintained by ancient and modern practitioners, particularly thofe of Doctors Cullen, Home, Rollo, Rutherford and Bree, he gives the following diagnofis:

"Though the etymology of the word diabetes may admit of every immoderate difcharge of urine being called diabetes, yet, by the definitions of a number of writers, it feems to be juftifiable to reject all thofe cafes of immoderate derate difcharges of urine, as diabetes, which are not accompanied with thirft, and to ftate the diagnoftics of this difeafe to be great thirft and fhrinking, and drynefs of the fkin, with increafe of faccharine or infipid-tafted urine. A great quantity of urine in diabetes is often obferved, when the thirft, fhrinking, and even faccharine urine have been detected. And, as there is reafon to believe, that the *phymofis** is often the only fymptom which has excited the attention of the patient, that fymptom, with the fenfation of heat in the urethra, on making urine, ought to be noticed in the nofological definition of this difeafe." pp. 52 and 53.

Dr. Girdelfton concludes with a "Poftcript," which we deem of fufficient importance to lay before our readers: "Since thefe fheets were printing, my friend, Dr. Lubbock, has detected two more cafes of diabetes, by the affection of the prepuce. One of thefe patients was firft an out-patient, and afterwards an in-one, in the Norwich hofpital. This patient was of about fifty years of age, and naturally of a lean habit: his gums were fore, his prepuce affected, his urine fweet, and its quantity, alternating with a difcharge by the bowels, varied from ten pints in the twenty-four hours to half a pint. Dr. Lubbock put the patient upon a diet of animal decoction, cold meat and milk, and gave him fmall dofes of opium, which plan mitigated the diabetic fymptoms. The Doctor tried alfo the hepatized ammonia, without being able to difcover from it any advantage. He alfo, at my requeft, ordered this patient one day a diet of river fifh, and the next day a whiting, or fea-fifh. But, contrary to the obfervations of my patient, very little alteration was obferved from the firft day's diet: and on the fecond day of eating the fifh, the quantity of urine was diminifhed. The patient on the fecond day had a motion extraordinary, fo that probably the diabetic fymptoms were rather increafed than diminifhed; though they were not fo evidently increafed by this diet, as in my patient. Dr. L. had intended to have omitted the milk in this patient's diet, and more rigidly to have confined him to animal food. But the temptation of drink, at the late Norwich Election, caufed the patient to defert from the hofpital, and prevented Dr. L. from being able to extend his experiments on this patient, or to learn any thing more about him. Dr. L. found in this cafe, as he had done in two former diabetic patients, that no fort of external application would relieve the phymofis, but that it was always more or lefs fevere, as the other diabetic fymptoms were increafed or diminifhed."—pp. 111 and 112.

---

*Practical Obfervations on the Cure of Wounds and Ulcers on the Legs, without reft; illuftrated with Cafes:* By Thomas Whately, Member of the Corporation of Surgeons of London. 8vo. 352 pp. (Price 7s.) *London.* Cadell and Davies; Johnfon; Callow.

We do not hefitate to recommend a work, which not only poffeffes much intrinfic merit, but has an additional claim to our notice, from the benevolent intention of the author. It is, within our knowledge, the firft publication dedicated to the Prefident, Vice-Prefidents, and Members of the

*Not long since (remarks the author), conversing with my friend Dr. Lubbock, of Norwich, he informed me that a person had called upon him to be relieved from a phymosis, which had troubled him for a few weeks, and for which he had been previously under the direction of a medical gentleman for some time; that, upon finding the phymosis did not yield to the applications commonly useful in such cases, Dr. Lubbock began to spspect it was connected with the diabetic diathesis, and upon inquiry found that the patient discharged eight pints of urine in twenty-four hours, sweet to the taste, and readily passing into the vinous fermentation; he was in apparent ealth, and hrd made no complaint of general disease:——And it is with Dr. Lubock's permission that this fact is published.

the Society for the relief of the Widows and Orphans of medical Men, in London and its Vicinity. The author hopes that, by expofing in a variety of inftances, the want of uniformity, and confequently the want of fuccefs, in the prevailing method of treating ulcerated legs, he may contribute fomething to the eafe and comfort of mankind; and folicits permiffion to appropriate the profits of this publication to that humane inftitution, the defign of which is, to raife a fund of £10,000, for the diftreffed families of our unfortunate brethren.

The following extracts from our author's Preface, will unfold his principles, as well as the practice he has adopted, and fufficiently enable the reader to judge of his merit.

"The efficacy of preffure (fays he) in counteracting the effects of the dependent pofture, was indeed known to the father of Englifh furgery; and the ufe of the laced ftocking was recommended by him for this purpofe; nor can there be any doubt, that the good effects of it in his hands were very manifeft. His ideas, however, feem not to have been much regarded by fucceeding furgeons. We find but little faid by the writers on furgery, on the effects of preffure in the cure of ulcers on the lower extremities, previous to the appearance of Dr. Underwood's treatife. Yet I am aware, that there always have been practitioners who were acquainted with the importance of this mode of treatment, and have adopted it in their practice. I had, myfelf, an opportunity of feeing the extraordinary fuccefs attending it, during my apprenticefhip in the country. It is matter of fact, however, that the practice is very far from being general. Even in one of the lateft publications on the fubject, and this too by a furgeon of the firft eminence, the effect of preffure is not much relied upon for the cure of thefe complaints. It is indeed there ftated, in feveral paffages, not only that no benefit is derived from compreffion in feveral fpecies of thefe ulcers, but that many ulcers are rendered worfe, more painful, and more unhealthy in their appearance by its ufe*. That there are certain conditions of an ulcer, which will not bear compreffion, I have allowed, and have endeavoured to point out the proper treatment, to bring on a fit ftate for the application of that preffure; but that an experienced furgeon fhould pafs over fo flightly this moft effential part of the cure, and even fpeak of it as *frequently* injurious, is a circumftance hardly to be attributed to any other caufe than that of a carelefs and ineffectual application of the bandages. For my own part, having now been for twenty years conftantly in the habit of treating a very large number of thefe cafes, I can fpeak fo confidently of the good effects of preffure, properly applied, that I can venture to affirm, that he who doubts its efficacy, has never given it a fair trial.

"In the cafes which are added to this Effay, very little variety of dreffing was ufed; the cure was almoft always trufted principally to the preffure made on the limb, under the exceptions particularly fpecified in the work. My fuccefs has been fo uniform, that I cannot but be anxious to fee this practice become eftablifhed, and generally followed. Nothing but a conviction, that in promoting this end, I am really doing an important fervice to my fellow-creatures, could have induced me to appear before the tribunal of the public, confcious as I am of my incompetency as a writer. But may I not hope, that the plain tale of a practical man will be heard, though not told with the graces of elegant language.

In whatever manner this attempt be received, I cannot doubt but that the

* See Home on Ulcers of the Legs.

the practice here recommended muſt, in the end, prevail, notwithſtanding it has this great obſtacle to contend with, that ſurgeons muſt condeſcend, for the moſt part, to apply the bandages with their own hands. The clumſy and ineffectual manner in which this buſineſs is too frequently done, can never be expected to produce the deſired effect. I am certain that if the neceſſary pains be taken, according to the directions here laid down, ſuch effects will uniformly follow, as muſt convince the unprejudiced mind, that to have recourſe to the operation of tying varicoſe veins, and the application of a great variety of remedies, can be *very rarely*, moſt probably *never* neceſſary. I can ſafely declare, that all ſuch caſes as are deſcribed by Mr. Home* to be cured by this operation, have readily yielded under the proper management of preſſure alone.

"Since theſe papers were preparing for the preſs, I have ſeen with pleaſure Mr. Baynton's new method of treating theſe complaints. Every thing that is there ſaid on the efficacy of his method, may be conſidered as confirming the doctrine laid down in the following pages. His mode however of making the preſſure with adheſive plaſter, appears to me inconvenient, and on ſeveral accounts objectionable. I have no doubt but that the proper application of compreſſes and flannel rollers, would, in every caſe recorded by him, have produced ſimilar good effects. The inſtances of ſucceſs by his method, after the ſuppoſed failure by the roller, I can only attribute to this, that the preſſure made with the plaſters was applied by his own hands, whereas that with the roller was, probably, as is uſual, ſo made that the effect intended by it could not poſſibly have been obtained. No ſurgeon, who will not be at the trouble of applying them himſelf, can be a judge of what may be effected by the proper management of the roller and compreſſes."

The volume before us is divided into ten diſtinct chapters, in which the ingenious author reſpectively treats of the difficulties attending the cure of wounds and ulcers on the legs; of the nature, treatment, and cure, peculiar to local wounds and ulcers, and ſuch as are connected with diſeaſes of the conſtitution; of eryſipelatous inflammation after wounds and ulcers of the legs; of the treatment of carious ulcers, &c.—After having ably commented upon the different methods of curing wounds and ulcers of the legs, namely, with and without reſt; and likewiſe explained the method of preventing relapſes, he has added *one hundred and ſixty-ſeven* ſhort caſes, all of which appear to have terminated ſucceſsfully, while the patients were permitted to walk about, and purſue their uſual occupations.

In a 'Note' ſubjoined to theſe caſes, Mr. Whately obſerves, "that about *one hundred and twenty* of theſe patients are *now* living, and perfectly well. About twenty of the remaining number are dead; and twenty-ſeven are removed to freſh places of abode."

For the information of thoſe readers who are not already in poſſeſſion of this uſeful book, we ſhall here inſert the formula of the calamine cerate, which the author has generally uſed, as it is not made according to the London Diſpenſatory. He obſerves, however, that this cerate is more apt to grow rancid than the common calamine cerate, and on that account is not ſo eligible for a plaſter; but, with this exception, it is a better compoſition, and leſs liable to evaporate than the latter. The following is the formula alluded to:

"Take

* See Home on Ulcers of the Legs.

"Take of fresh hog's lard, three pounds;
Fresh litharge plaster, one pound and a half;
Calamine prepared, one pound.
"Mix them together according to art, into a calamine cerate.

"To this formula I shall add another for making a cerate, which nearly resembles the unguentum tripharmacum of the old Dispensatory, but being less oily, it makes a much more adhesive plaster. It should be spread on rag, or silk, as an external covering to the dressing on lint, where a tow plaster cannot be conveniently used; as in wounds of the face or hands, a bubo, or any other sore, where an external plaster cannot be readily retained in its situation by a bandage. This plaster is likewise so mild that it never irritates the skin. I have found it also a very useful plaster in fractures. The following is the formula;

"Take of fresh litharge plaster, one pound;

Fresh hog's lard, six ounces;

Vinegar, four ounces;

"Mix them together according to art into an ointment."

Besides those before-mentioned, the author has annexed nine other "Cases of carious ulcers on the legs," accompanied with a coloured plate, and ample explanations. These cases also convey much novel and interesting matter, especially relative to the *internal* exfoliation of bones, and are well deserving the attention of every chirurgical practitioner.

We cannot conclude our account of this practical work, without making the reader acquainted with the hints and directions contained in the '*Postscript*,' which appear to us so precise and useful, that we shall quote them in the author's own words; for the additional reason, as he here likewise replies to the objections made against the application of the roller, by Mr. Baynton, of whose Essay, on the same subject, we propose to give some account in the next article.

"Although" says Mr. Whately, "there are many observations made in the body of the foregoing work respecting the proper method of applying the roller and compresses, it perhaps may not be unacceptable to the younger part of the profession to add a few more particulars on so important a matter.

"I have said, that the flannel rollers should be four inches wide, to allow for shrinking in washing; by which I would have it understood, that when they are made of that width, they are a little too wide; especially for those whose legs are small. The best width for a flannel roller designed for those who have slender legs, is three inches; but for those whose legs are of a large size, they should be always three inches and a half in width. They must therefore be torn at first a little wider, that they may be of their proper width when repeatedly washed. It will likewise be found, that rollers made of fine, soft, and open flannel, will answer much better than those made of coarse or hard flannel.

"For those who have full-sized legs, the length of six yards is but just sufficient to answer all the purposes intended by a roller; but in those who have very small legs, five yards is a sufficient length. Care should be taken that the rollers be washed in very hot water; and they should be hung up to dry immediately on being washed. If these precautions be not attended to, repeated washing them will, in some kinds of flannel, make them as narrow as tape, by which they will be rendered almost useless. They should be often washed, as they are much softer, and of course sit easier, when quite clean, than when they are soiled."

Desciptive

[To be resumed in our next Number.]

*Descriptive Account of a new Method of treating old Ulcers of the Legs:* By THOMAS BAYNTON, Surgeon, of Bristol. The second edition, enlarged, corrected, and considerably improved. 8vo. 152 pp. 1799. *London,* Hurst.

In our first volume, pp. 186 and fol. we have duly noticed the first edition of Mr. BAYNTON's Essay on this subject, and have given the outline of his method of treating old ulcers of the legs, by bringing the edges of ulcers nearer together by means of adhesive plasters, and added a general account of the circumstances and reflections which induced him to adopt the essential and successful improvement in that department of chirurgical practice At present, therefore, we shall furnish our readers with a more minute description of the method peculiar to the author.

"I shall now endeavour," says he, "to describe the means whereby these advantages are obtained; and as it will be perceived that there is little more in the materials recommended than surgeons have been long in the habit of using, it must also be perceived that the difference in the effects are to be ascribed to the *manner* in which those materials are applied. Success therefore depending upon the *mode* of their application, I shall be more particular in my description of *it* than perhaps may to many appear necessary; but being convinced that almost every thing which can be desired may be obtained in such cases, if the principles are kept in view, and a proper application of the means persevered in, I hope by the fulness of my description to spare those who adopt the plan, the inconveniences and disappointments which may be experienced, if the steadiest attention does not direct its application.

"The parts should be first cleared of the hair sometimes found in considerable quantities upon the legs, by means of a razor, that none of the discharges, by being retained, may become acrid and inflame the skin, and that the dressings may be removed with ease at each time of their renewal, which in some cases where the discharges are very profuse, and the ulcers very irritable, may perhaps be necessary twice in the twenty-four hours, but which I have in every instance been only under the necessity of performing once in that space of time.

"The plaster should be prepared by slowly melting, in an iron ladle, a sufficient quantity of litharge plaster or diachylon, which if too brittle when cold to adhere, may be rendered adhesive by melting half a drachm of resin with every ounce of the plaster; when melted, it should be stirred till it begins to cool, and then spread thinly upon slips of smooth porous calico of a convenient length and breadth, by sweeping it quickly from the end held by the left hand of the person who spreads it, to the other, held firmly by another person, with the common elastic spatula used by apothecaries; the uneven edges must be taken off, and the pieces cut into slips about two inches in breadth, and of a length that will, after being passed round the limb, leave an end of about four or five inches. The middle of the piece so prepared, is to be applied to the sound part of the limb opposite to the inferior part of the ulcer, so that the lower edge of the plaster may be placed about an inch below the lower edge of the sore, and the ends drawn over the ulcer with as much gradual extension as the patient can well bear, other slips are to be secured in the same way, each above and in contact with the other, until the whole surface of the sore and the limb are completely covered, at least one inch below, and two or three above the diseased part.

"The whole of the leg should then be equally defended with pieces of soft calico three or four times doubled, and a bandage of the same about three inches in breadth, and four or five yards in length, or rather as much as will be sufficient to support the limb from the toes to the knee, should be applied applied as ſmoothly as can be poſſibly performed by the surgeon, and with as much firmneſs as can be borne by the patient, being paſſed firſt round the leg at the ancle joint, then as many times round the foot as will cover and ſupport every part of it except the toes, and afterwards up the limb till it reaches the knee, obſerving that each turn of the bandage ſhould have its lower edge ſo placed as to be about an inch above the lower edge of the fold next below.

After having giuen an ingenious explanation of the *modus operandi* of this new method of curing inveterate ulcers of the legs, the author examines the different opinions of other practitioners, which appear to him more or leſs unſatisfactory, and corroborates the mode of practice he has adopted with eighteen ſuccesſful caſes.

We find the teſtimonies of Meſſrs. Everard Home, Thomas Henry, William Simmons, R. Sandford, and Thomas Shute, all of whom have, in public and private practice, introduced the uſe of adheſive plaſters to ulcerated legs: Mr. Home, in particular, allows that it is one of the greateſt improvements which has been made in that branch of ſurgery; and in many caſes of private patients; and that its ſucceſs has anſwered his moſt ſanguine expectation. Mr. H. however, principally alludes to caſes of *long ſtanding*; for in thoſe he found it the moſt effectual: there are many ulcers too irritable to admit of it, and theſe of courſe require a different mode of treatment.

We do not pretend to decide, whether Mr. Whately's method of employing rollers, or Mr. Baynton's practice of uſing ſtrips of adheſive plaſters, in general, deſerves the preference: we are rather inclined to think that each of theſe methods has its peculiar advantages, which muſt be aſcertained by the judicious practitioner, according to the particular caſes and conſtitutions of the patient.

[*Want of room has obliged us to diſcontinue the quotations from Mr. Baynton's treatiſe, in the preſent Number, but we ſhall ſupply that deficiency in our next.*]

